# Quorum sensing: cell-to-cell communication in *Saccharomyces cerevisiae*

**DOI:** 10.3389/fmicb.2023.1250151

**Published:** 2023-11-23

**Authors:** Linbo Li, Yuru Pan, Shishuang Zhang, Tianyou Yang, Zhigang Li, Baoshi Wang, Haiyan Sun, Mingxia Zhang, Xu Li

**Affiliations:** ^1^School of Life Sciences and Technology, Henan Institute of Science and Technology, Xinxiang, Henan, China; ^2^Hainan Key Laboratory of Tropical Microbe Resources, Institute of Tropical Bioscience and Biotechnology, Chinese Academy of Tropical Agricultural Sciences, Haikou, Hainan, China

**Keywords:** quorum sensing, quorum sensing molecules, quorum sensing system, response mechanism, *Saccharomyces cerevisiae*

## Abstract

Quorum sensing (QS) is one of the most well-studied cell-to-cell communication mechanisms in microorganisms. This intercellular communication process in *Saccharomyces cerevisiae* began to attract more and more attention for researchers since 2006, and phenylethanol, tryptophol, and tyrosol have been proven to be the main quorum sensing molecules (QSMs) of *S. cerevisiae*. In this paper, the research history and hotspots of QS in *S. cerevisiae* are reviewed, in particular, the QS system of *S. cerevisiae* is introduced from the aspects of regulation mechanism of QSMs synthesis, influencing factors of QSMs production, and response mechanism of QSMs. Finally, the employment of QS in adaptation to stress, fermentation products increasing, and food preservation in *S. cerevisiae* was reviewed. This review will be useful for investigating the microbial interactions of *S. cerevisiae*, will be helpful for the fermentation process in which yeast participates, and will provide an important reference for future research on *S. cerevisiae* QS.

## 1 Introduction

The cell-to-cell interactions that microorganisms use to communicate and coordinate their social behavior are crucial when they live in a community. Quorum sensing (QS) is a cell-to-cell signaling mechanism observed in microbe populations that is density-dependent (Efremenko et al., 2023). As the number of microbial cells reaches a critical level, it sends signals to neighboring microorganisms through signaling molecules called quorum sensing molecules (QSMs) that alter their behavior by regulating the expression of certain genes (Padder et al., 2018). The QSMs in microbial cells are related with the regulation of secondary metabolite production, symbiosis, bioluminescence, synthesis of antibiotics, regulation of nitrogen-fixing genes, pathogenesis, morphogenesis, competence, biofilm formation, etc (Chen et al., 2002; An et al., 2014). Historically, *Saccharomyces cerevisiae* has been used in food production and food processing for a long time, and it is generally used as a potential microbial cell factory for producing a variety of chemicals due to it being a very important type of strain. Research on QS of *S. cerevisiae* has been paid more and more attention since 2006, especially after the QSMs in *S. cerevisiae*, phenylethanol (Phe-OH) and tryptophol (Try-OH), and tyrosol (Tyr-OH) were identified (Chen and Fink, 2006; Kebaara et al., 2008; Albuquerque and Casadevall, 2012).

In this review, we present research history, and hotspots of QS in *S. cerevisiae*, focusing particularly on the underlying mechanism of the QS system for *S. cerevisiae*. In addition, we also introduce the application of *S. cerevisiae* QS and QSMs in the food fermentation process. This review offers insights into density-dependent cell-to-cell communication of *S. cerevisiae*, which will be helpful for controlling the fermentation process in which *S. cerevisiae* is involved, such as beer brewing, wine making, and alcoholic fermentation.

## 2 Milestones and hotspots of QS research

### 2.1 Milestones of QS research

Quorum sensing (QS) is a process that links gene expression to the cell density of microbial populations (Albuquerque and Casadevall, 2012; Borea et al., 2018). During growth and reproduction, microbial populations secrete and release some specific signaling molecules due to the increase in cell density, and the concentration of their secreted signaling molecules can reach a threshold when the cell density reaches a certain level, and the signaling molecules then strongly induce synchronized gene expression by binding to their homologous receptor proteins, thus initiating or shutting down specific population biological behaviors (Turan et al., 2017; Franco et al., 2021; Tian et al., 2021). QS occurs widely in many different bacteria, as well as some fungi and yeast (Jagtap et al., 2020). QS was first identified and studied in bacteria, subsequently, fungal QS has been reported, and since 2006, QS in *S. cerevisiae* has attracted increasing attention.

In 1968, the behavior now identified as QS was discovered in the bioluminescent gram-negative bacterium *Photobacterium fischeri*, which exhibited bioluminescence upon reaching a high population density (Kempner and Hanson, 1968). In 1969, Lingappa et al. (1969) first reported the regulation of fungal QS, that is, filamentous growth of the human fungal pathogen *Candida albicans* depending on cell density. It was not until 1994 that Fuqua et al. (1994) named this density-dependent self-regulation phenomenon QS and defined the minimum behavioral unit needed to elicit certain bacterial behaviors at high cell densities as a “quorum” of bacteria. Since Chen and Fink (2006) first identified Phe-OH and Try-OH as active QSMs that induce filamentous growth in *S. cerevisiae* in 2006, QS in *S. cerevisiae* has attracted great attention. Subsequently, Smukalla et al. (2008) first proposed in 2008 that the flocculation of *S. cerevisiae* is regulated by QS. In 2013, Zupan et al. (2013) established an effective approach to study QS in yeast fermentation, which contributed to the separation, detection, and quantification of the potential QSMs Phe-OH, Try-OH, and Tyr-OH. Their work was followed in 2015 by Avbelj et al. (2015), which monitored the kinetics of QSM production and ARO genes expression in *S. cerevisiae* during wine fermentation, and initially confirmed correlations between peak production rates of the monitored QSMs Phe-OH, Try-OH, and Tyr-OH and peak expression of the genes responsible for their synthesis, i.e., *ARO8*, *ARO9*, and *ARO10*. Lenhart et al. (2019) studied the variation in the filamentous growth of an environmental strain of *S. cerevisiae* and its response to QSMs in 2019, and their research showed that the filamentous growth phenotype induced by QSMs was species-specific and only a few strains showed a response. In 2021, Huang and Reardon (2021a) demonstrated for the first time that adding yeast QSMs to reduce cell growth in *S. cerevisiae* could increase ethanol yield. In the same year, data from Najmi and Schneider (2021) suggested that yeast may use potential QS mechanisms to regulate ribosome biogenesis, particularly rRNA synthesis. Unlike other studies of morphological switching, this is the first QS mechanism to target a central metabolic process in yeast, providing a research basis for its potential therapeutic value. In 2022, Winters et al. (2022) proposed that Phe-OH induced filamentous growth could not be considered a QS mechanism because it did not meet the previously defined “physiological concentration” requirement. Still, Britton et al. (2023) challenged his view in the latest study. The study points out that the physiological concentrations reported by Winters et al. (2019) do not reflect the natural occurrence observed in a diffusion-limited fermentation environment using industrially relevant strains. Tian et al. (2023) studied the effect of the addition of QSMs on the fermentation of *S. cerevisiae*, and showed that the exogenous addition of Phe-OH promoted the accumulation of ethanol, which was similar to the results of Huang and Reardon (2021a) and provided basic data for using QSMs more than antibiotics in the prevention of contamination during the industrialized bioethanol production. QS has been observed in many microbial species, regulating the many diverse processes, including secretion of virulence factors, bioluminescence, cell adhesion and elongation, population density control, motility, biofilm formation, cell morphology and dimorphism, sporulation and antibiotic production (Miller and Bassler, 2001; Albuquerque and Casadevall, 2012; Wongsuk et al., 2016; Schuster et al., 2017; Padder et al., 2018; Gomez-Gil et al., 2019).

### 2.2 Hotspots of QS research

**FIGURE 1:**
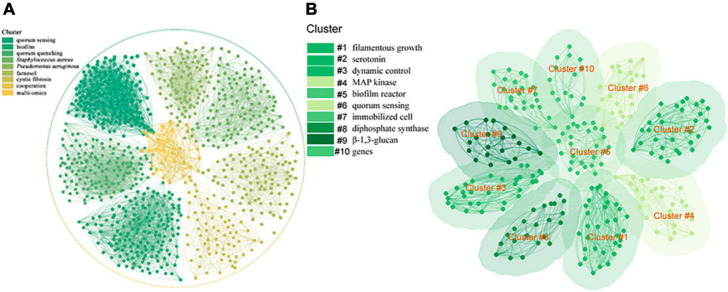
**(A)** Hotspots of QS research in microbiology **(B)** hotspots of QS research in *S. cerevisiae*.

The importance of studying QS has received great attention, and since 1994, a good number of research publications on QS have been published. We searched the QS-related literature in the Web of Science (WOS) database and obtained 8,823 research articles, and used CiteSpace software (6.3. R3) to analyze their bibliometrics and visualization (Chen and Song, 2019). By keyword co-occurrence mapping, it was found that the main QS-related research topic has been quorum sensing, biofilm, quorum quenching, *Staphylococcus aureus*, *Pseudomonas aeruginosa*, farnesol, cystic fibrosis, cooperation, and multi-omics (Figure 1A). Bibliometric analysis allowed us to understand the global context of QS (Ashraf et al., 2021). The effect of the QS system on biofilm formation and regulation has been found, and research on quorum quenching is being undertaken and is a hotspot in related fields. At the same time, QS and disease prevention have always been the focus of researchers, and pathogenic bacteria, such as *S. aureus* and *P. aeruginosa*, are important biomaterials for QS research. Farnesol is one of the QSMs that is given much attention. With the development of multi-omics technology in recent years, an increasing number of omics technologies have been applied to QS research.

To gain a clear view of QS in *S. cerevisiae*, we also used the search terms “*S. cerevisiae*” and “QS” to search the articles in the WOS database, retrieved 97 research articles, and analyzed their bibliometrics and performed visualization by CiteSpace software (Chen and Song, 2019). The keyword co-occurrence mapping showed that the main QS-related research topics for *S. cerevisiae* were filamentous growth, serotonin, dynamic control, MAP kinase, biofilm reactor, quorum sensing, immobilized cell, diphosphate synthase, β-1,3-glucan, and genes (Figure 1B). Filamentous growth has been shown to be a response to QS mechanisms in fungi through morphological transitions between single cells and pseudohyphae (Britton et al., 2023). Serotonin has been shown to be a compound produced during the metabolism of aromatic amino acids in *S. cerevisiae* and has an effect on yeast growth and cell morphology (Beatriz et al., 2018; Gonzalez, 2021). Dynamic control can be implemented either through a pathway dependent circuit or a pathway independent circuits (Yang et al., 2021; Xu et al., 2023). QS provides a pathway independent approach to autonomous dynamic control for the synthetic biology of microorganisms, where once critical signal concentrations are reached, population-wide cellular responses are initiated to influence gene expression to achieve simultaneous optimization of systems involving QS communication, cell growth competition, and cooperative production. This is more environmentally friendly and operable than chemical synthesis in industry (Dinh et al., 2020; Ge et al., 2020). The highly conserved mitogen-activated protein kinase (MAPK) signaling pathway is critical for eukaryotic cells to make appropriate adaptive responses to environmental changes (Gonzalez, 2021). Indeed, one of the best-known model systems for this signaling pathway is the response of haploid *S. cerevisiae* cells to mating pheromones, where QSMs stimulate yeast cell filamentous growth primarily via the MAPK pathway (Gomez-Gil et al., 2019; Seibel et al., 2021). Biofilm formation is one of the main regulatory mechanisms of QS. Phe-OH has a positive effect on biofilm formation of *S. cerevisiae* (Zhang et al., 2021b). As a barrier, biofilm can provide a stable internal environment for yeast cells to cope with the adverse environment such as ethanol stress and nutrient insufficiency (Tian et al., 2023). In the production of fuel ethanol, the conversion rate of repeated batch fermentation of *S. cerevisiae* in biofilm reactor is higher than that of free fermentation. In addition to a high yield of ethanol, a short fermentation cycle and excellent tolerance to ethanol were observed during this fermentation process (Yang et al., 2019; Zhang et al., 2021b). Immobilized cell technology is a new adsorption method developed based on microbial QS (Chen et al., 2020). After the cells are adsorbed on the carrier, a large number of cells grow, forming a dense bacterial community, and then showing QS (Yang et al., 2018). Utilizing immobilized cell technology can significantly improve the conversion of biomass, contributing to novel strategies to achieve the Sustainable Development Goals (Yang et al., 2019; Huang and Reardon, 2021b). Farnesol diphosphate synthetase is an important protein in the process of QSM farnesol synthesis. Using population density-regulated protein degradation system to dynamically regulate the expression level of related genes is a promising method for metabolic pathway control (Yang et al., 2021). β-1,3-glucan is the main component of fungal cell wall and is mainly responsible for regulating cell morphology endowing osmotic stability, and preventing stress in yeast (Mould and Hogan, 2021; Seibel et al., 2021). When QSMs accumulate to a certain threshold, they trigger synchronous gene expression by binding to their homologous receptor proteins, thus inducing QS (Britton et al., 2023; Efremenko et al., 2023). These data, when analyzed, showed that the available knowledge on the subject is still limited, and very little attention has been given to QS in *S. cerevisiae* and the mechanism of action of its QSMs, which has hindered the exploration of *S. cerevisiae* QS mechanism and the development of promising applications. Therefore, the study of QS in *S. cerevisiae* and the application of its QSMs in controlled fermentation have been the focus of attention in recent years.

## 3 QS system of *S. cerevisiae*

### 3.1 Regulation mechanism of QSMs synthesis in *S. cerevisiae*

**FIGURE 2:**
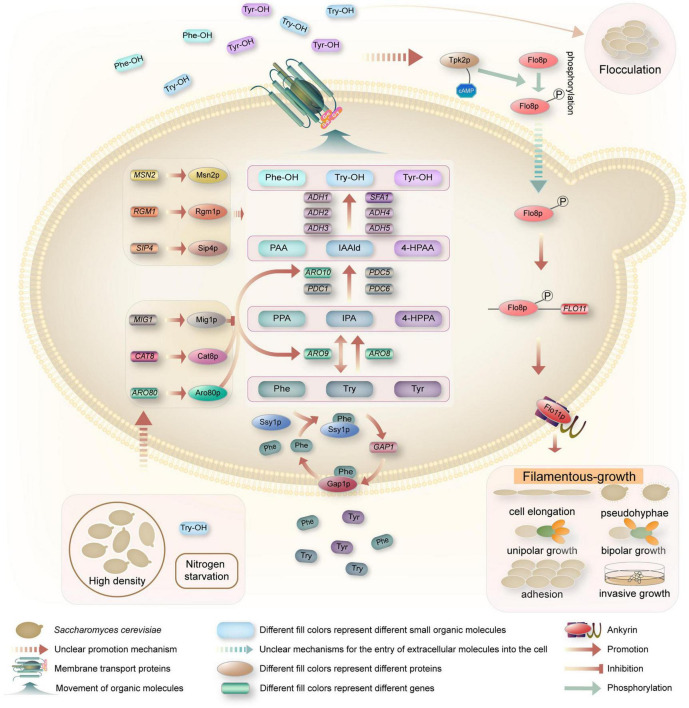
Schematic illustrations of quorum-sensing system in *S. cerevisiae*. When yeast cells reached quorum, the expression of *GAP1* and the recovery of Gap1p activity were induced by the amino acid osmotic factor Ssy1p, and the aromatic amino acids were transported into the cells. Phe, Try, and Tyr were transaminated to PPA, IPA, and 4-HPPA under the catalysis of aromatic amino acid transaminase encoded by *ARO8* and *ARO9* genes, respectively. These α-keto acids are then decarboxylated by *ARO10*, *PDC1*, *PDC5*, and *PDC6* encoded decarboxylase to produce PAA, IAA-ld, and 4-HPAA. These higher aldehydes are then reduced to Phe-OH, Try-OH, and Tyr-OH under dehydrogenase encoded by the dehydrogenase genes *ADH1*, *ADH2*, *ADH3*, *ADH4*, *ADH5*, and *SFA1*. Aromatic alcohols are transported outside the cell as QSMs by transporters, accumulating to a certain threshold and triggering QS phenotypes, i.e., flocculation or filamentous growth. Phe-OH and Try-OH affect the up-regulation of *FLO11* via the cAMP-dependent PKA subunit Tpk2p and the transcription factor Flo8p. Flo11p, the product of *FLO11*, is necessary for filamentous growth and is involved in cell elongation, pseudomycelium growth, unipolar growth, bipolar growth, cell adhesion, and invasive growth.

In *S. cerevisiae*, the QSMs Phe-OH, Try-OH, and Tyr-OH are synthesized from the corresponding amino acids phenylalanine (Phe), tryptophan (Try) and tyrosine (Tyr) by transamination, decarboxylation and reduction via the Ehrlich pathway in a low-nitrogen environment (Tan and Prather, 2017; Figure 2). When only aromatic amino acids are present as nitrogen sources, *GAP1* expression is induced, and the general amino acid permease Gap1p restores transport activity. In addition, the amino acid osmotic factor Ssy1p is a sensor via which cells detect extracellular aromatic amino acids as signals (Chen et al., 2016). Taking Phe as an example, Ssy1p first receives the Phe signal in a low nitrogen environment, which induces the expression of *GAP1* and the restoration of Gap1p activity. Gap1p transports Phe into the cell and participates in metabolic activity (Iraqui et al., 1999; Chen et al., 2017). In the Ehrlich pathway, Phe-OH is transaminated from Phe to the α-keto acid phenylpyruvate (PPA), which is decarboxylated to the higher aldehyde phenylacetaldehyde (PAA), which is then reduced to the higher alcohol Phe-OH by alcohol dehydrogenase (Etschmann et al., 2002). Via a similar mechanism, Try is transaminated to indole-3-pyruvate (IPA), followed by carboxylation to indole-3-acetaldehyde (IAA-ld), and then reduced to Try-OH (Chen and Fink, 2006; Avbelj et al., 2015). Tyr is transaminated to 4-hydroxyphenylpyruvate (4-HPPA), decarboxylated to 4-hydroxyphenylacetaldehyde (4-HPAA), and then reduced to Tyr-OH (Mas et al., 2014).

The transamination step in the Ehrlich pathway is catalyzed by the aromatic amino acid aminotransferase. There are two isoenzymes in *S. cerevisiae*, namely, aromatic amino acid aminotransferases I and II, both of which belong to the first family of aminotransferases and are encoded by the *ARO8* and *ARO9* genes, respectively, and have broad substrate specificity in the Ehrlich pathway. There are four known enzymes in *S. cerevisiae* that can catalyze the production of α-keto acids to higher aldehydes, which are encoded by *ARO10*, *PDC1*, *PDC5*, and *PDC6* (Brion et al., 2014; Choo et al., 2018). These enzymes participate in complex interactions with each other for processes such as activation, inhibition, and coordination. Among them, the PPA decarboxylase encoded by *ARO10* is the main enzyme involved in decarboxylation, and its broad substrate specificity has been confirmed by various genetic methods (Dickinson et al., 2003; Chen et al., 2017). The last step of the Ehrlich pathway for conversion to aromatic alcohols can be catalyzed by various alcohol dehydrogenases (Hazelwood et al., 2008). Six dehydrogenase genes are involved in the catalytic process in *S. cerevisiae*, including the five alcohol dehydrogenase genes *ADH1*, *ADH2*, *ADH3*, *ADH4*, and *ADH5* and the formaldehyde dehydrogenase gene *SFA1*. Any of these genes can individually catalyze the final step of the Ehrlich pathway, the dehydrogenation of aldehydes to form higher alcohols (Hazelwood et al., 2008).

As a crucial pathway to synthesize aromatic alcohols, regulation of the Ehrlich pathway is very important, however, the regulatory mechanism is not clear. With the development of bioinformatics and the study of the yeast response to aromatic alcohols, a few regulators that might be related to the Ehrlich pathway have been predicted. For example, Chen and Fink (2006) demonstrated that the transcription factor Aro80p controls the expression of Ehrlich pathway genes in response to aromatic amino acids (Figure 2). Aro80p, a member of the Zn2Cys6 protein family, was identified as a transcriptional activator for the induction of transaminase and decarboxylase genes by aromatic amino acids, which can activate the expression of the ARO9 and ARO10 genes by binding to ARO9 and ARO10 promoter binding sites (Iraqui et al., 1999; Lee and Hahn, 2013; Chen et al., 2017). Subsequently, Wuster and Babu (2010) predicted that Cat8p, Mig1p, Sip4p, Rgm1p, and Msn2p might be key transcriptional regulators controlling the differential expression of the genes affected by aromatic alcohols in their study of transcriptional control of the QS response in yeast. Cat8p is a zinc cluster protein as well as a regulator that represses the expression of the ADH2 gene in *S. cerevisiae* (Wang et al., 2017). Similarly, Mig1p is a transcription factor with two Cys2His2 zinc finger motifs, and it was reported that Mig1p regulates CAT8 and depends on the release of Mig1p from the promoter of CAT8 (Schüller, 2003). Furthermore, through multiple transcriptional network analysis methods, Wuster and Babu (2010) proposed that Cat8p and Mig1p participate in QS and might be important for aromatic alcohol-mediated communication. This was confirmed by Wang et al. (2017), who reported that a CAT8 overexpression strain and a MIG1 deletion strain could promote the expression of ARO9 and ARO10, and both could increase the production of Phe-OH. How CAT8 and MIG1 induce gene expression and whether they affect the performance of yeast have not yet been elucidated.

### 3.2 Factors influencing the production of QSMs in *S. cerevisiae*

The production of QSMs in *S. cerevisiae* depends on environmental factors, including cell density (Avbelj et al., 2015, 2016), nitrogen content (Chen and Fink, 2006), ethanol (Avbelj et al., 2015), and aerobic/anaerobic growth conditions (Avbelj et al., 2016; Jagtap et al., 2020; Franco et al., 2021). These factors contribute to the production of QSMs, which trigger phenotypic changes to promote morphological adaptations to new environmental conditions.

In *S. cerevisiae*, cell density regulates QSMs production. The production of QSMs per yeast cell is greater in yeast cells at high population density than in yeast cells at low population density (Zupan et al., 2013). When cells reach their specific quorum, the expression of *ARO9* and *ARO10* genes is upregulated, which stimulates the production of aromatic alcohols (Chen and Fink, 2006; Avbelj et al., 2015; Figure 2). Try-OH activates the transcription factor Aro80p and the expression of transaminase and decarboxylase genes, thus resulting in a positive feedback loop (Wuster and Babu, 2010; Jagtap et al., 2020). Nitrogen content in the medium affects QSMs production (Cullen and Sprague, 2001, 2012; Chen and Fink, 2006), as high concentrations of ammonium inhibit the expression of transaminases, decarboxylases, and dehydrogenases required for the synthesis of aromatic alcohols. It was reported that aromatic alcohol production peaked when the ammonium concentration was less than or equal to 50 μM and was significantly reduced at concentrations greater than 500 μM. The product ethanol during *S. cerevisiae* fermentation was also demonstrated to negatively influence the overall rate and onset time of aromatic alcohol synthesis and inhibit cell growth (Avbelj et al., 2015). However, it is unclear whether this reduction is related to the inhibition of aromatic alcohol synthesis or whether the cell density is not reaching the quorum due to ethanol inhibition. The study showed that when 2% ethanol was added to the medium, the cell growth of *S. cerevisiae* did not change, and the production kinetics of Try-OH and Tyr-OH were weakly inhibited compared with the control without ethanol. With the addition of 6% ethanol, there was a significant delay in peak production of all three aromatic alcohols, most likely due to weak inhibition of cell growth. When 8% ethanol was added, the inhibitory effect on cell growth was enhanced, resulting in complete inhibition of Try-OH production, while the expression of the other two aromatic alcohols was delayed and only weakly expressed, possibly because the cell concentration did not exceed a specific threshold for initiating Try-OH production. When 12% ethanol was added, no cell growth was observed and no aromatic alcohols were produced. The report also showed that in all cases where ethanol was added, despite differences in cell growth curves, when the cell concentration reached 2 × 10^7^ ∼ 3 × 10^7^ cells/mL, 3 × 10^7^ ∼ 4 × 10^7^ cells/mL, and 2 × 10^7^ ∼ 4 × 10^7^ cells/mL, the production of Phe-OH, Try-OH, and Tyr-OH still began. These concentrations appear to represent the quorum for the synthesis of each of the three aromatic alcohols under the conditions of fermentation by this yeast (Avbelj et al., 2015). Aerobic/anaerobic conditions affect the production of aromatic alcohols, and alcohol dehydrogenase genes are upregulated under anaerobic conditions, which is beneficial for the production of aromatic alcohols by *S. cerevisiae*. Studies have shown that *S. cerevisiae* converts Phe to a mixture of 90% Phe-OH and 10% phenylacetate under aerobic conditions, while anaerobic conditions result in the almost complete conversion of Phe to Phe-OH (Vuralhan et al., 2003). Meanwhile, Avbelj et al. (2016) assert that *S. cerevisiae* produces higher concentrations of aromatic alcohol when grows in anaerobic conditions than in aerobic conditions.

### 3.3 Response mechanism of QSMs in *S. cerevisiae*

To date, many studies have investigated the response mechanism of Phe-OH and Try-OH, while the response mechanism of Tyr-OH has rarely been reported. The sensing of Phe-OH and Try-OH relies on the cyclic adenosine monophosphate (cAMP)-dependent protein kinase A (PKA) subunit Tpk2 (Figure 2). It has been reported that the levels of cAMP are critical determinants of cellular filamentous growth (Mosch and Fink, 1997), regulate the activity of a family of protein kinases, referred to as PKA, and Tpk2, the catalytic subunit of PKA, promote filamentous growth (Pan and Heitman, 1999, 2002; Cullen and Sprague, 2012). Phosphorylation of Flo8p by Tpk2 leads to Flo8p activation (Pan and Heitman, 1999; Guo et al., 2000; Reynolds and Fink, 2001; Chen and Fink, 2006; Cullen and Sprague, 2012), and activated Flo8p, as a transcriptional activator, binds to regions of the *FLO11* promoter to promote gene expression (Pan and Heitman, 2002). Flo11p, a product of *FLO11*, is a glycosylated phosphatidylinositol (GPI)-anchored cell surface flocculating protein that is required for filamentous growth and is involved in pseudohyphal growth, invasive growth, unipolar growth, bipolar growth, cell elongation, and cellular adhesion (Smukalla et al., 2008; Avbelj et al., 2015; Winters et al., 2019).

Filamentous growth, which usually occurs under nutrient starvation, is thought to be a mechanism that allows cells to grow in their surroundings and forage for available nutrients (Jagtap et al., 2020) and is characterized by elongated cell morphology, unipolar budding, partial separation of mother and daughter cells, and substrate incursion (Gimeno et al., 1992). Depending on the cell type, filamentous growth in *S. cerevisiae* can be described as pseudohyphal or invasive growth. Filamentous growth in diploid cells has been described as pseudohyphal growth involving elongated cell morphology and alterations in budding patterns. A similar but separate response in haploid cells is known as haploid invasive growth, as these cells form filaments, which penetrate the agar (Cullen and Sprague, 2001, 2012). Preliminary studies by Chen and Fink (2006) have demonstrated that QSMs Phe-OH and Try-OH stimulated pseudohyphal growth under nitrogen-limited starvation conditions. Additionally, recent studies have investigated the effects of exogenous aromatic alcohols on the transformation of *S. cerevisiae* from the yeast cell form to a filamentous cell form. González et al. (2018) demonstrated that aromatic alcohols were able to affect invasive and pseudohyphal growth in a manner dependent on nutrient availability. However, Lenhart et al. (2019) demonstrated that the filamentous phenotypic response induced by Phe-OH and Try-OH in *S. cerevisiae* may be a strain-specific effect by using computational image analysis to quantify the production of pseudohyphae and assaying the diverse 100-genome collection of environmental isolates. Britton et al. (2023) then studied the native variation of yeast-to-filamentous phenotypic transition and its induction by Phe-OH in commercial brewing strains was investigated. The results showed that phenotypic switching was a general, highly variable response that occurred only in selected brewing strains. The results again confirmed that the filamentous phenotypic response induced by aromatic alcohol may be a strain specific effect. However, it is still necessary to accurately understand the internal molecular mechanisms and related metabolic pathways of QSMs in response to the differences in yeast filamentous growth phenotypes. As noted by Britton et al. (2023), it is possible that there are several reasons to explain the differences between commercially produced strains, such as that some strains may not be able to sense Phe-OH, or that some others may sense Phe-OH but not initiate the response, or that some strains may be unable to undergo filamentation. In addition, the author believes that it is necessary to study the effects of QS or QSMs on the taste, color, number of viable cells, ethanol production, physical and chemical properties, and nutrition and healthcare value of commercial brewing wine.

Flocculation is a social feature that depends on multiple cells cooperating simultaneously and a cooperative protection mechanism that protects cells from stressful environments, and the flocculation behavior of *S. cerevisiae* was shown to be related to Try-OH (Smukalla et al., 2008). Smukalla et al. (2008) found that adding the QSMs Try-OH could induce strong flocculation in the diploid strain *S. cerevisiae* EM93. In their study, the effects of Phe-OH, Try-OH, Tyr-OH, ethanol, butanol, and isoamyl alcohol on the flocculation behavior of EM93 were investigated, and it was found that this strain did not flocculate in nutrient-rich medium. This flocculation behavior is suggested by these authors to be a protective mechanism mediated by Try-OH, where mutual adhesion leads to the formation of flocs that prevent diffusion and thus provide physical protection for the innermost cells from harmful compounds in the external environment. Since flocculence requires a fairly large microbial population and Flo adhesin expression is only functional at higher population densities, it stands to reason that this particular adaptive feature, flocculation behavior in *S. cerevisiae*, occurs in cells exposed to Try-OH (Britton et al., 2020).

## 4 Biotechnological application of QS in *S. cerevisiae*

### 4.1 Adaptation to stress

Filamentous growth stimulated by *S. cerevisiae* QSMs is a foraging response that favors yeast survival under stressful conditions (Silva et al., 2007). Because both Flo8p and Mss11p are LisH domain-containing transcription factors and function as heterodimers to regulate *FLO11* gene expression (Su et al., 2009), improved the tolerance of yeast to butanol by increasing the expression of the filamentous growth response pathway transcription factor Mss11p. In addition, aromatic alcohols also stimulate other transcription factors, such as Mig1p and Cat8p, to regulate different stress responses so that high-density yeast fermentation based on QS could show strong tolerance and catalytic activity (Douglas et al., 2008). In addition, previous studies have shown that there is a relationship between QS and reactive oxygen species (ROS)-related metabolism in *S. cerevisiae*, where *ARO80* has been identified as the key gene regulating QS (Kang et al., 2021). This finding was confirmed by Kang et al. (2022), who reported that deletion of the *ARO80* gene could decrease the oxidative tolerance of yeast cells and that adding exogenous QSMs contributed to increasing the oxidative tolerance of the mutant. Furthermore, the specific sensing of N-(3-oxododecanoyl)-L-homoserine lactone (C_12_) from the opportunistic human pathogen *Pseudomonas aeruginosa* by *S. cerevisiae* activated its general stress response to reduce the damage caused by strong oxidative stress (Delago et al., 2021). Meanwhile, Ren et al. (2016) demonstrated that long-chain C_12_-HSL, an N-acyl homoserine lactone (AHL) of bacterial QSMs, could increase the ethanol tolerance of *S. cerevisiae*.

Studies on ethanol tolerance by QS in *S. cerevisiae* have only been reported in recent years. A related investigation demonstrated that purified Tyr-OH (50 μM) exhibited protective effects against *S. cerevisiae* under 12% ethanol stress, and the fermentation capacity was significantly improved (Nath et al., 2021b). In addition, it has been reported that QS plays a certain role in the resistance to heavy metal stress. Nath et al. (2021a) conducted research on the effect of the QSMs Tyr-OH on heavy metal tolerance in *S. cerevisiae*. The tolerance to the heavy metals zinc (Zn^2+^), manganese (Mn^2+^), cobalt (Co^2+^), and copper (Cu^2+^) was determined in the presence or absence of Tyr-OH. They found that under the influence of Tyr-OH, the strain showed strong growth ability at the upper limit of heavy metal tolerance, was more adaptable to stressful environments, and had a protective effect. Compared with the use of physical and chemical means, it is more feasible and safe to use microbial QS system to face the stressed environment and enhance their own protection.

### 4.2 Increasing fermentation product formation

Phe-OH, Try-OH, and Tyr-OH, as QSMs, have been demonstrated to be synthesized and secreted by *S. cerevisiae* and have been used to improve fermentation products, including for enhancement of aroma in foods and drinks (Etschmann et al., 2002; Wang et al., 2011) and improvement of wine flavor and quality (Gonzalez-Marco et al., 2010). studied the antifungal activity mediated by Picha spp. QSMs Phe-OH and its effect on ethanol metabolism during the fermentation of sauce-flavor baijiu. Metabolome analysis and amplicon sequencing along with metatranscriptomic analysis demonstrated that Phe-OH can mediate antifungal mechanisms, including protein synthesis and DNA damage, and its role in food fermentation. The study can promote the niche establishment and growth of the functional yeast, the improvement of ethanol metabolism, and the enhancement of wine flavor (Zhang et al., 2021c). In addition, it has been also demonstrated that the QSM Try-OH has an effect on regulating ethanol fermentation in wine fermentation (José et al., 2018). At the same time, QSMs have also been shown to be associated with increased alcohol production from fermentation products. Some studies have provided evidence that the ethanol yield can be improved by adding yeast QSMs to reduce the cell growth of *S. cerevisiae* and observed that ethanol production can be up to 15% higher (Huang and Reardon, 2021a). Tian et al. (2023) not only determined the concentration of QSMs in *S. cerevisiae* but also investigated the effect of exogenous *S. cerevisiae* QSMs on the sole fermentation of *S. cerevisiae* and co-fermentation of *S. cerevisiae* with *Lactobacillus plantarum*. The results showed that the concentration of QSMs produced by *S. cerevisiae* increased with the increase in cell density, and only Phe-OH promoted the ethanol production of *S. cerevisiae*. The ethanol concentration of the sole fermentation of *S. cerevisiae* loaded with 120 mg/L Phe-OH reached 3.2 g/L in 9 h, which was 58.7% higher than that of the group without Phe-OH addition.

Luo et al. (2018) applied a novel adsorption method developed based on QS, biofilm immobilization, to rapidly enrich yeast, produce a large amount of biofilm, and achieve high levels of alcohol fermentation with an alcoholic strength of up to 15.3% (v/v). Huang and Reardon (2021b) immobilized *S. cerevisiae* in alginate and incorporated it into a two-column immobilized cell reactor system. In addition, the yeast QSM Phe-OH is added to increase ethanol production by restricting growth and diverting sugar to ethanol. Zhang et al. (2021a) used acetic acid as a signaling molecule to initiate a QS system to promote the production of 2,3-butanediol in *S. cerevisiae* W141. The results showed that the yield of 2,3-butanediol was proportional to the cell density, and when 1.5 g/L acetic acid was added during the fermentation process, the yield of 2,3-BD was the highest, reaching 3.01 ± 0.04 g/L.

Moreover, the QS system is used as a tool in metabolic engineering. Recently, the design, construction, and implementation of QS circuits for the programmed control of bacterial phenotypes and metabolic pathways have attracted much attention (Ge et al., 2020). Williams et al. (2015) constructed a gene switch that automatically triggered expression at high population density in *S. cerevisiae* and used this strategy to increase the production of para-hydroxybenzoic acid produced by the shikimate pathway. Yang et al. (2021) used a QS-mediated protein degradation system to increase α-farnesene production in *S. cerevisiae*. First, they integrated a plant hormone cytokinin system with the endogenous yeast Ypd1-Skn7 signal transduction pathway and designed QS-regulated protein degradation circuits for dynamic metabolic pathway control in *S. cerevisiae*. Then, based on this control method, they constructed an auxin-induced protein degradation system and applied this circuit to control the degradation of Erg9 to produce α-farnesene, and the titer of α-farnesene increased by 80%. In addition, the QS system of yeast has also been applied in the field of sewage treatment, and biofilm augmentation in yeast-based microbial fuel cells. These results demonstrate the feasibility of novel strategies to achieve sustainability goals in biomass conversions.

### 4.3 Food preservation

Phe-OH, Try-OH, and Tyr-OH can be used as antioxidants, antimicrobials, and disinfectants (Cueva et al., 2012). These aromatic alcohols have been used as biotechnological tools in the food industry, fragrances, and cosmetics due to their antimicrobial and oxidative protection properties (Gonzalez-Marco et al., 2010; Lu et al., 2016). The most common way to prevent spoilage in the food industry is the use of chemical preservatives. However, the use of chemicals not only is harmful to the environment and leads to unnecessary residues in food but also is bad for health. In this regard, Phe-OH produced by yeast can be advantageously applied in food as a natural preservative.

Liu et al. (2014) found that the yeast *K. apiculata* strain 34-9 acted as an antagonist with biological control activity against postharvest diseases of citrus fruit because the strain could produce Phe-OH, which could influence filamentous growth, adhesion, and biofilm formation and biological control efficiency. Likewise, Phe-OH produced by *Pichia anomala* was shown to inhibit spore germination, growth, toxin production, and gene expression in *Aspergillus flavus* (Hua et al., 2014; Chang et al., 2015). Huang et al. (2020) studied the antifungal effects of the QSMs Phe-OH against the food spoilage molds *Penicillium expansum* and *Penicillium nordicum*. The results showed that Phe-OH inhibited the conidial germination and growth of the two molds in a non-lethal, reversible, and concentration-dependent manner, but unlike other antifungal agents, phenethyl alcohol did not damage the conidial cell membrane. Compared with the chemical synthesis method, the biosynthesis method of Phe-OH, Try-OH, and Tyr-OH is more environmentally friendly and feasible to achieve green sustainable development.

## 5 Conclusion

In this review, we first introduced the definition of QS and selected the main research milestones for QS from 1968 to 2023, especially those for *S. cerevisiae* since 2006. This helped us recognize the research history and understand the important events in QS research. The research articles on the QS of *S. cerevisiae* in the last two decades are indexed in WOS and were visually analyzed based on CiteSpace software. The statistical results provide an important reference for future research. In this paper, the QS system of *S. cerevisiae* was discussed, with emphasis on the regulation mechanism of QSMs synthesis, the influencing factors of QSMs production, and the response mechanism of QSMs. The importance of understanding the mechanisms will help evaluate the cellular interactions of *S. cerevisiae* in terms of the effects on QS signal producers and receivers. Finally, the biotechnological application of QS in *S. cerevisiae* is introduced, mainly in the aspects of adaptation to stress, increasing the formation of fermentation products, and food preservation. If anything, reviewing the QS and QSMs in *S. cerevisiae* will be useful for investigating microbial interactions and controlling industrial fermentation processes such as brewing and food production.

At present, the research on QS of microorganisms including yeast is still in the initial stage, and there are still many problems to be solved. For example, in commercial brewing, it is necessary to discuss the role of QS of yeast in mixed fermentation and its influence on enhancing flavor substances or nutrients, so as to improve the ecological niche of brewing wine. In the field of biofuels, it is necessary to explore the influence of QS systems on microbial fermentation to produce fuel ethanol, fuel cells, etc., in order to improve the rate of energy self-sufficiency. In the field of biological control, the QS system is used to increase the effect of biological control, strengthen biological remediation, improve environmental protection, and realize the new strategy of sustainable development. In the field of medicine and health, the effects of QS systems as a new tool on food spoilage, fungal infection, clinical environment, and disease treatment were discussed. Putting all together, it will be of great significance to continue researching and developing the application of QS systems.

## Author contributions

LL, YP, and SZ carried out the original literature screening under the supervision of ZL, TY, and MZ. YP drafted the manuscript with inputs from LL, SZ, and TY. All authors contributed to the article, edited, revised and approved the final version of the manuscript.
